# Survival Prediction in Patients With Bladder Cancer Undergoing Radical Cystectomy Using a Machine Learning Algorithm: Retrospective Single-Center Study

**DOI:** 10.2196/86666

**Published:** 2026-02-19

**Authors:** Francesco Andrea Causio, Vittorio De Vita, Andrea Nappi, Melissa Sawaya, Bernardo Rocco, Nazario Foschi, Giuseppe Maioriello, Pierluigi Russo

**Affiliations:** 1 Italian Society for Artificial Intelligence in Medicine (SIIAM - Società Italiana Intelligenza Artificiale in Medicina), Rome, Italy; 2 University Department of Life Sciences and Public Health, Section of Hygiene, Università Cattolica del Sacro Cuore, Largo F Vito 1, Rome, 00168, Italy, 39 0630154396; 3 Computer Science Department, University of Twente, Twente, The Netherlands; 4 Université Paris-Saclay, UVSQ, Inserm, Gustave Roussy, CESP, Villejuif, France; 5 University Department of Urology, Università Cattolica del Sacro Cuore, Rome, Italy; 6 University Department Medicine and Translational Surgery, Università Cattolica del Sacro Cuore, Rome, Italy; 7 Department of Life Science, Health, and Health Professions, Università Degli Studi Link, Rome, Italy

**Keywords:** cystectomy, disease-free survival, artificial intelligence, neoplasm staging, retrospective studies, urinary bladder neoplasms, clinical decision-making, machine learning, statistical models

## Abstract

**Background:**

Traditional statistical models often fail to capture the complex dynamics influencing survival outcomes in patients with bladder cancer after radical cystectomy, a procedure where approximately 50% of patients develop metastases within 2 years. The integration of artificial intelligence (AI) offers a promising avenue for enhancing prognostic accuracy and personalizing treatment strategies.

**Objective:**

This study aimed to develop and evaluate a machine learning algorithm for predicting disease-free survival (DFS), overall survival (OS), and the cause of death in patients with bladder cancer undergoing cystectomy, using a comprehensive dataset of clinical and pathological variables.

**Methods:**

Retrospective data of 370 patients with bladder cancer who underwent radical cystectomy at Fondazione Policlinico Universitario Agostino Gemelli IRCCS, Rome, Italy, were collected. The dataset comprised 20 input variables, encompassing demographics, tumor characteristics, treatment variables, and inflammatory markers. For specific analyses and models, we used patient subcohorts. The CatBoost algorithm was used for regression tasks (DFS in 346 patients, OS in 347 patients) and a binary classification task (tumor-related death in 312 patients). Model performance was assessed using mean absolute error (MAE) for regression and *F*_1_-score for classification, prioritizing a minimum recall of 75% for tumor-related deaths. Five-fold cross-validation and Shapley additive explanations (SHAP) values were used to ensure robustness and interpretability.

**Results:**

For DFS prediction, the CatBoost model achieved an MAE of 18.68 months, with clinical tumor stage and pathological tumor classification identified as the most influential predictors. OS prediction yielded an MAE of 17.2 months, which improved to 14.6 months after feature filtering, where tumor classification and the systemic immune-inflammation index (SII) were most impactful. For tumor-related death classification, the model achieved a recall of 78.6% and an *F*_1_-score of 0.44 for the positive class (tumor-related deaths), correctly identifying 11 of 14 cases. Bladder tumor position was the most influential feature for cause-of-death prediction.

**Conclusions:**

The developed machine learning algorithm demonstrates promising accuracy in predicting survival and the cause of death in patients with bladder cancer after cystectomy. The key predictors include clinical and pathological tumor staging, systemic inflammation (SII), and bladder tumor position. These findings highlight the potential of AI in providing clinicians with an objective, data-driven tool to improve personalized prognostic assessment and guide clinical decision-making.

## Introduction

In the evolving landscape of health care, the integration of artificial intelligence (AI) into clinical decision-making has gained significant momentum, particularly in the realm of oncology [1,2]. With advancements in machine learning techniques, health care professionals are increasingly harnessing the power of AI to enhance diagnosis, prognosis, and treatment planning. The exponential growth of digital health care data, including electronic health records, medical imaging, genomic data, and real-time patient monitoring, has fueled the development of predictive algorithms [1,3].

The field of urology is complex: cancerous conditions benefit from the leverage of additional data sources and decision-making algorithms that allow physicians to plan treatment while considering several complex factors. Urological cancers, including prostate, bladder, and renal cancers, place a considerable burden on health care systems worldwide [4]. These malignancies often require complex management involving early diagnosis, accurate staging, and personalized treatment strategies to optimize patient outcomes. Traditional methods of assessing prognosis rely heavily on statistical models that may not capture the multifaceted nature of cancer behavior and patient responses to treatment. Conventional regression statistics often fail to provide the depth of analysis required to address the complexities of cancer management. In contrast, AI techniques, such as artificial neural networks, Bayesian networks, and neuro-fuzzy modeling systems, offer innovative approaches to constructing data-driven models that can adapt to the heterogeneous nature of cancer [5].

The potential of AI in predicting patient outcomes is particularly evident in its ability to analyze large datasets without the constraints of predetermined statistical distributions. By leveraging retrospective data, we can develop algorithms that not only identify patterns and correlations but also provide insights into individual patient behavior. This capability is crucial for clinicians who face the challenge of tailoring treatment plans to the unique characteristics of each patient. In the context of mortality and postoperative survival, the application of AI can provide critical insights that enhance our understanding of patient outcomes following surgical interventions. The ability to predict which patients are at higher risk of complications or recurrence can lead to more informed clinical decisions, ultimately improving the quality of care [6]. For instance, machine learning algorithms can analyze a multitude of variables, including clinical, pathological, and demographic factors, to generate individualized risk profiles that guide treatment strategies and follow-up plans [7]. Recent urological research has shown that combining hematological inflammation indexes with machine learning algorithms can improve the prediction of surgical outcomes, as demonstrated in patients who underwent urethroplasty [8].

In this study, we focus specifically on the training of an AI algorithm using retrospective data collected from patients diagnosed with bladder cancer who underwent radical cystectomy. Patients with localized muscle-invasive or recurrent non–muscle-invasive bladder cancer benefit most from radical cystectomy, which may be preceded by neoadjuvant chemotherapy in selected cases, in terms of local disease control. Even with sufficient local control achieved through cystectomy, approximately 50% of patients develop metastases within 2 years and may ultimately die from the disease. This is likely due to the existence of regional or distant microscopic metastatic disease at the time of surgery [9]. The proposed methodology will involve the comprehensive examination of variables associated with patient demographics, tumor characteristics, treatment modalities, and postoperative outcomes. Using machine learning techniques, we aim to identify key predictors of mortality and postoperative survival, ultimately constructing a predictive model with potential relevance for clinical decision-making.

## Methods

### Study Design

We collected retrospective data on patients with high-risk and very high-risk non–muscle-invasive bladder cancer and muscle-invasive bladder cancer who underwent radical cystectomy at Fondazione Policlinico Universitario Agostino Gemelli IRCCS in Rome, Italy. The dataset included data on various clinical and pathological variables from 370 patients.

### Ethical Considerations

Ethical approval was obtained from the institutional ethical review board (protocol number 676‐02). As primary consent for data collection covered secondary analyses, additional consent was not required for this study. The data used in this study were anonymized, and no compensation was provided to patients.

### Data Collection and Preprocessing

Clinical and pathological data were extracted from medical records, including demographic, lifestyle, tumor, treatment, and laboratory variables. The dataset was split into three outcome-specific subsets to maximize the usable sample for each task:

1. DFS dataset: predicting disease-free survival (DFS) in months (346 patients in total).
2. OS dataset: predicting overall survival (OS) in months (347 patients in total).
3. Death cause dataset: for classification purposes, the classes “death from other causes” and “alive” were merged into a single negative class to create a binary variable. Therefore, the cause is defined as either “no” or “yes,” depending on whether it was tumor related (312 patients in total).

The variables included in the dataset are detailed in Table 1. All categorical variables were cast as strings to allow native handling by CatBoost (version 1.2.8; Yandex).

A total of 20 input variables were selected for model development.

**Table 1.** Variables included in the study

Variable (English)	Description	Data type	Input/output
Patient demographics and lifestyle			
AGE	Age (years)	Numerical	Input
BMI	Body mass index (kg/m^2^)	Numerical	Input
SEX	Biological sex (0: man, 1: woman)	Categorical	Input
SMOKE	Smoker (0: no, 1: yes)	Categorical	Input
Patient medical history			
DM	Patient has diabetes mellitus (0: no, 1: yes)	Categorical	Input
PRIOR SURGERY	Patient had previously undergone surgery in the abdominal area (0: no, 1: yes)	Categorical	Input
PRIOR RADIOTHERAPY	Patient had previously received radiotherapy in the abdominal area (0: no, 1: yes)	Categorical	Input
PRIOR SYSTEMIC CHEMOTHERAPY	Patient had previously received systemic chemotherapy (0: no, 1: yes)	Categorical	Input
Tumor characteristic			
BLADDER TUMOR POSITION	Identifier of tumor position (0: intertrigonal zone, 1: right periosteal, 2: left periosteal, 3: dome, 4: posterior wall, 5: right lateral wall, 6: left lateral wall, 7: prostatic urethra, 8: anterior wall, 9: entire bladder, 10: bladder base)	Categorical	Input
TUMOR DIMENSION	Tumor dimension (cm)	Numerical	Input
PRE-HYDRONEPHROSIS	Hydronephrosis (0: no, 1: right hydronephrosis, 2: left hydronephrosis, 3: bilateral hydronephrosis)	Categorical	Input
H.E. TURV	Histological examination for transurethral resection of the bladder (0: localized to mucosa +/– submucosa multirecurrent, 1: muscle-invasive, 2: squamous)	Categorical	Input
LVI	Lymphovascular invasion (0: absent, 1: present)	Categorical	Input
CTS	Clinical tumor stage (0: cTa, 1: cTis, 2: cT1, 3: cT2, 4: cT3, 5: cT4)	Categorical	Input
TC	Tumor classification (1: T0, 2: Ta, 3: Tis, 4: T1, 5: T2a, 6: T2b, 7: T3a, 8: T3b, 9: T4a, 10: T4b)	Categorical	Input
Inflammatory and immune marker			
SII	Systemic immune-inflammation index (decimals)	Numerical	Input
Treatment and outcome			
UD	Urinary diversion type (0: Bricker ileal conduit, 1: ureterocutaneostomy, 2: vesicoileal pouch)	Categorical	Input
RECURRENCE	Tumor recurrence (0: no, 1: yes)	Categorical	Input
DFS	Disease-free survival after treatment (in months)	Numerical	Output
OS	Overall survival: time from diagnosis/treatment start to death from any cause (in months)	Numerical	Output
DEATH CAUSE	Cause of death (X: alive, 1: other, 2: cancer); later merged (0: alive + other, 1: cancer)	Categorical	Output

### Machine Learning Models

To predict clinical outcomes, we used the CatBoost algorithm for both regression (DFS and OS) and classification (cause of death) tasks, as it is effective for small and structured datasets.

For DFS and OS, we applied CatBoostRegressor models. For predicting the cause of death, we used the CatBoostClassifier, with the binary outcome of death being tumor related or not.

### DFS and OS Model Evaluation

For the regression tasks (DFS and OS), we evaluated model performance using mean absolute error (MAE) to quantify the average prediction error in months.

### Cause-of-Death Classification Model

For the classification task (cause of death), we evaluated performance using the *F*_1_-score. The *F*_1_-score is a single metric that balances precision and recall, particularly useful in cases of imbalanced classes where the positive class is of primary interest. For class 1 (tumor-related deaths), it was calculated as the harmonic mean of precision and recall:


(1) F1=2∗(Precision∗Recall)/(Precision+Recall)

Precision is the proportion of correctly identified positive predictions among all positive predictions, and recall is the proportion of correctly identified positive predictions among all actual positives.


(2) Precision=TP/(TP+FP)

(3) Recall=TP/(TP+FN)

Confusion matrices were used to examine prediction distributions, and probability thresholds were adjusted to optimize recall while limiting false positives. To account for class imbalance in the classification task, we applied custom class weights. We adjusted the decision threshold, aiming for a minimum recall of 75% to ensure that most tumor-related deaths were accurately identified and classified.

### Cross-Validation and Hyperparameter Tuning

All models were trained and evaluated using 5-fold cross-validation to ensure generalizability and reduce the risk of overfitting, especially given the relatively small dataset. In addition, we applied early stopping with a patience range of 30 to 50 rounds, allowing the model to terminate training once performance ceased to improve on the validation fold.

To enhance the interpretability and transparency of the developed machine learning models, we used violin plots and Shapley additive explanations (SHAP) scatterplots to investigate the impact of variables on the prediction of the results. Violin plots show the effect of each variable on the results, both in terms of direction (favorable or unfavorable) and intensity. The SHAP scatterplot assigns an importance value to each feature for a particular prediction. For each patient, the SHAP values revealed the specific features driving the predicted risk of tumor death. By aggregating the SHAP values across the entire cohort, the overall impact and importance of each clinical and pathological variable on the model’s outcome predictions were determined. This enabled the identification of the most significant factors influencing the outcomes of patients with bladder cancer after cystectomy.

This paper presents only the most significant results of the analysis. The complete analysis is available online for open access [10].

### Transparent Reporting of a Multivariable Prediction Model for Individual Prognosis or Diagnosis Guidelines for AI

To enhance the transparency, interpretability, and reproducibility of our machine learning-based prediction models, this study adheres to the Transparent Reporting of a Multivariable Prediction Model for Individual Prognosis or Diagnosis (TRIPOD) statement, specifically considering the extensions for AI (TRIPOD+AI). The TRIPOD+AI guidelines provide a standardized framework for reporting studies that develop or validate prediction models, ensuring that sufficient detail is provided for critical appraisal and replication by other researchers. By following these guidelines, we aim to clearly articulate the study design, data characteristics, model development process, and performance evaluation, thereby contributing to the responsible and rigorous application of AI in medical research (Table 2).

**Table 2.** Transparent Reporting of a Multivariable Prediction Model for Individual Prognosis or Diagnosis checklist for reporting studies involving artificial intelligence (TRIPOD+AI).

TRIPOD+AI item	Description of reporting in this study
1. Title	Survival Prediction in Patients with Bladder Cancer Undergoing Radical Cystectomy Using a Machine Learning Algorithm: Retrospective Single-Center Study
2. Abstract	The abstract summarizes the study's objectives, methods, key findings, and conclusions.
3. Introduction - background	The introduction will establish the clinical context of bladder cancer, the prognostic challenges, and the rationale for using machine learning.
4. Methods - participants	
4a. Eligibility criteria	Patients who underwent radical cystectomy for bladder cancer, with available data for selected variables
4b. Settings and locations	Data collected retrospectively from a single institution: Fondazione Policlinico Universitario Agostino Gemelli IRCCS, Rome, Italy
4c. Source of data	Patient medical records
5. Data acquisition method	Retrospective data extraction into a spreadsheet
6. Methods - outcome	
6a. Definition of outcomes	Disease-free survival (DFS): time in months from treatment to recurrence or death (event) or last follow-up (censored) Overall survival (OS): time in months from diagnosis/treatment start to death from any cause (event) or last follow-up (censored) DEATH CAUSE: binary classification (0: did not die from the tumor, 1: died from the tumor)
6b. Outcome measurement	DFS and OS were calculated from documented dates. The cause of death was extracted from medical records and recategorized for binary classification.
7. Methods - predictors	
7a. Definition of all predictors	AGE: patient’s age (years) BMI (kg/m^2^) DM: patient has diabetes mellitus (0: no, 1: yes) PRIOR SURGERY: patient had previously undergone surgery in the abdominal area (0: no, 1: yes) PRIOR RADIOTHERAPY: patient had previously received radiotherapy in the abdominal area (0: no, 1: yes) PRIOR SYSTEMIC CHEMOTHERAPY: patient had previously received systemic chemotherapy (0: no, 1: yes) BLADDER TUMOR POSITION: identifier of tumor position (0: intertrigonal zone, 1: right periosteal, 2: left periosteal, 3: dome, 4: posterior wall, 5: right lateral wall, 6: left lateral wall, 7: prostatic urethra, 8: anterior wall, 9: entire bladder, 10: bladder base) TUMOR DIMENSION: tumor dimension (cm) PRE-HYDRONEPHROSIS: pretreatment hydronephrosis (0: no, 1: right hydronephrosis, 2: left hydronephrosis, 3: bilateral hydronephrosis) SEX: biological sex (0: man, 1: woman) SMOKE: patient smokes (0: no, 1: yes) H.E. TURV: histological examination for transurethral resection of the bladder (0: localized to mucosa +/– submucosa multirecurrent, 1: muscle-invasive, 2: squamous) SII: systemic immune-inflammation index (decimals) UD: urinary diversion type (0: Bricker ileal conduit, 1: ureterocutaneostomy, 2: vesicoileal pouch) LVI: lymphovascular invasion (0: absent, 1: present) CTS: clinical tumor stage (0: cTa, 1: cTis, 2: cT1, 3: cT2, 4: cT3, 5: cT4) TC: tumor classification (1: T0, 2: Ta, 3: Tis, 4: T1, 5: T2a, 6: T2b, 7: T3a, 8: T3b, 9: T4a, 10: T4b)
7b. Predictor measurement	Predictors were measured clinically (eg, age and BMI), derived from patient history (eg, prior surgeries and smoking status), or derived from laboratory or pathology reports (eg, SII, tumor dimension, LVI, CTS, and TC).
8. Methods - sample size	
8a. Sample size determination	The available retrospective data determined the sample size. No formal power calculation was performed due to the exploratory nature of the study and the limitations of the data.
9. Methods - data handling	
9a. Handling of missing data	Rows with null values in specific critical variables (“TUMOR DIMENSION,” “LVI,” “TC,” “H.E. TURV,” “RECURRENCE,” “DFS,” “OS,” “DEATH CAUSE”) were removed. No imputation was performed.
9b. Data transformation	Numerical variables were type-adjusted to int or float. Categorical variables were explicitly converted to category type. “DEATH CAUSE” was recategorized into a binary format.
10. Methods - model development	
10a. Model type	CatBoostRegressor (for DFS and OS) and CatBoostClassifier (for DEATH CAUSE).
10b. Candidate predictors	All 17 selected independent variables were used as candidate predictors for each model, based on the relevant dataset (df1 [DFS], df2 [OST], df3 [DEATH CAUSE]).
10c. Handling of continuous predictors	Continuous predictors (AGE, BMI, TUMOR DIMENSION, SII) were used directly by CatBoost, which handles them internally.
10d. Handling of categorical predictors	Categorical predictors were identified and explicitly converted to string type before training. CatBoost natively handles categorical features without explicit one-hot encoding.
10e. Details of model fitting	CatBoostRegressor (iterations=1000, learning_rate=0.05, depth=6, loss_function=”RMSE,” eval_metric=”MAE,” early_stopping_rounds=50, random_seed=42, verbose=0). Similar configurations for CatBoostClassifier, with “Logloss” or “MultiClass” as loss function.
10f. Internal validation method	Data were split into training (80%) and testing (20%) sets using train_test_split with random_state=42. Five-fold cross-validation (KFold, shuffle=True, random_state=42) was performed on the training set.
10g. Performance metrics	Regression (DFS, OS): mean absolute error (MAE) Classification (DEATH CAUSE): *F*_1_-score for class 1 (tumor-related deaths), prioritizing recall
11. Assessment of prediction performance	Performance was assessed on the independent test set. For classification, a confusion matrix was used.
12. Model interpretation methods	CatBoost's built-in feature importance was used. Shapley additive explanations (SHAP) values were computed and visualized using violin plots and scatterplots to understand the individual contributions of each feature.

## Results

### Patient Characteristics

The study included a final cohort of 374 patients. After excluding incomplete records, the analytical sample sizes were 346 for DFS prediction, 347 for OS prediction, and 312 for death cause prediction. Some records indicate fewer than 374 patients, as not all characteristics were available for every individual in the population.

Table 3 presents the baseline clinical, pathological, and demographic characteristics of the study population, comprising 79.4% (297/374) men and 20.6% (77/374) women. A majority, 79.4% (296/373) were active smokers, and 21.4% (80/374) had a diagnosis of diabetes mellitus. Prior surgery was reported for 33.7% (126/374) of patients, while previous radiotherapy and systemic chemotherapy were less common, 5.1% (19/374) and 3.5% (13/374), respectively. Preoperative hydronephrosis was present in approximately one-third of cases, most frequently unilaterally.

**Table 3.** Characteristics of the patients in the dataset.

Characteristic	Value
Continuous variables	
Age (years), mean (SD)	75.2 (9.5)
BMI (kg/m^2^), mean (SD)	26.6 (4.2)
Tumor dimension (cm), median (IQR)	2.2 (1.1‐2.8)
SII^a^, median (IQR)	654.7 (408.0‐1047.0)
DFS^b^ (months), median (IQR)	23.0 (6.0‐52.8)
OS^c^ (months), median (IQR)	29.0 (10.8‐55.4)
Categorical variables, n/N (%)	
Sex	
Men	297/374 (79.4)
Women	77/374 (20.6)
Smoking status	
No	77/373 (20.6)
Yes	296/373 (79.4)
Diabetes mellitus	
No	294/374 (78.6)
Yes	80/374 (21.4)
Prior surgery	
No	248/374 (66.3)
Yes	126/374 (33.7)
Prior radiotherapy	
No	355/374 (94.9)
Yes	197/374 (5.1)
Prior chemotherapy	
No	361/374 (96.5)
Yes	13/374 (3.5)
Pre-hydronephrosis^d^	
None	257/374 (68.7)
Right	44/374 (11.8)
Left	40/374 (10.7)
Bilateral	33/374 (8.8)
Histological examination (H.E. TURV)	
Localized	138/372 (37.1)
Muscle-invasive	212/372 (57)
Squamous	22/372 (5.9)
Urinary diversion type	
Bricker ileal conduit	278/373 (74.5)
Ureterocutaneostomy	36/373 (9.7)
Vesicoileal pouch	59/373 (15.8)
Lymphovascular invasion	
Absent	158/371 (42.6)
Present	213/371 (57.4)
Clinical tumor stage	
cTa	132/366 (36.1)
cTis	43/366 (11.7)
cT1	77/366 (21)
cT2	74/366 (20.2)
cT3	31/366 (8.5)
cT4	9/366 (2.5)
Tumor classification	
T0	16/342 (4.7)
Ta	18/342 (5.3)
Tis	60/342 (17.5)
T1	47/342 (13.7)
T2a	54/342 (15.8)
T2b	7/342 (2)
T3a	80/342 (23.4)
T3b	10/342 (2.9)
T4a	42/342 (12.3)
T4b	8/342 (2.4)
Bladder tumor position	
Intertrigonal zone	44/365 (12.1)
Right periosteal	25/365 (6.8)
Left periosteal	39/365 (10.7)
Dome	22/365 (6)
Posterior wall	58/365 (15.9)
Right lateral wall	71/365 (19.5)
Left lateral wall	58/365 (15.9)
Prostatic urethra	8/365 (2.2)
Anterior wall	26/365 (7.1)
Entire bladder	12/365 (3.3)
Bladder base	2/365 (0.5)
Cause of death	
Alive	205/363 (56.5)
Other	71/363 (19.6)
Cancer	87/363 (23.9)

a SII: systemic immune-inflammation index.

b DFS: disease-free survival.

c OS: overall survival.

d Pre-hydronephrosis: pretreatment hydronephrosis.

Histologically, 57% (212/372) of tumors were muscle invasive, while 37.1% (138/372) were localized to the mucosa or submucosa, and 5.9% (22/372) exhibited squamous features. The most common urinary diversion method was Bricker ileal conduit (278/373, 74.5%), followed by vesicoileal pouch construction (59/373, 15.8%) and ureterocutaneostomy (36/373, 9.7%). Lymphovascular invasion was observed in 57.4% (213/371) of patients.

In terms of staging, the most frequent clinical tumor stages were cTa (132/366, 36.1%) and cT1 (77/366, 21%), while advanced stages (cT3 and cT4) were less common (40/366, 11%). Tumor classification was heterogeneous, with T3a (80/342, 23.4%) and Tis (60/342, 17.5%) being most prevalent.

Regarding tumor location, the most frequent sites were the right lateral wall (71/365, 19.5%), the posterior wall (58/365, 15.9%), and the left lateral wall (58/365, 15.9%). At the time of data collection, of 363 patients, 205 (56.5%) were alive, 87 (24%) had died due to cancer-related causes, and 71 (19.6%) had died from other causes.

### DFS Prediction

The CatBoostRegressor model was trained to predict DFS in months. Input variables are indicated in Table 1. After 5-fold cross-validation and manual hyperparameter tuning, the model achieved an MAE of 18.68 months, indicating that, on average, the model’s predictions deviated by approximately 1.5 years from the observed DFS.

**Figure 1.**
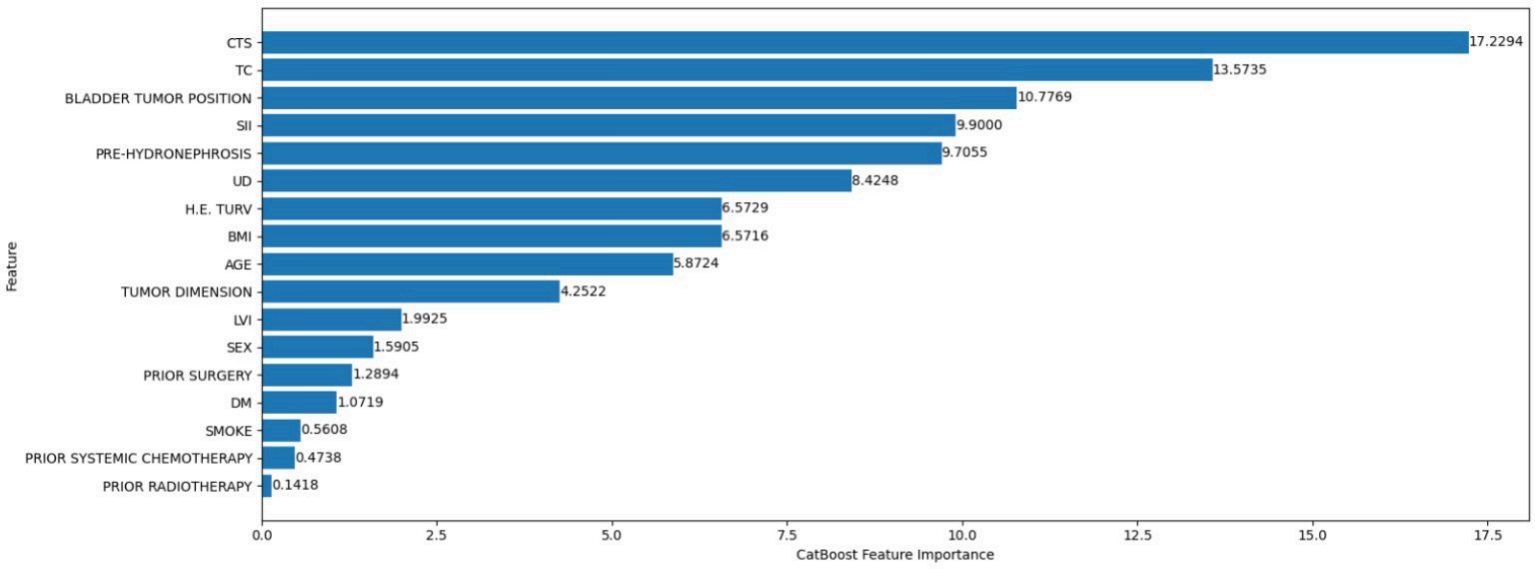
Global feature importance ranking to predict disease-free survival. CTS: clinical tumor stage; DM: diabetes mellitus; H.E. TURV: histological examination for transurethral resection of the bladder; LVI: lymphovascular invasion; PRE-HYDRONEPHROSIS: pretreatment hydronephrosis; SII: systemic immune-inflammation index; TC: tumor classification; UD: urinary diversion type*.*

Figure 1 presents the global feature importance ranking from the CatBoost model trained to predict DFS. This ranking reflects the contribution of each variable to reducing the model’s prediction across all patients. The most influential predictor was the clinical tumor stage, with an importance score of approximately 17, followed by the pathological tumor classification, with an importance score of approximately 14, reflecting the role of tumor invasiveness, local extension, and accurate tumor staging in DFS prediction. The systemic immune-inflammation index (SII) ranked highly, with a score of approximately 9.5. To a lesser extent, demographic and anatomical variables, such as BMI, age, and tumor dimension, also contributed to the model.

**Figure 2.**
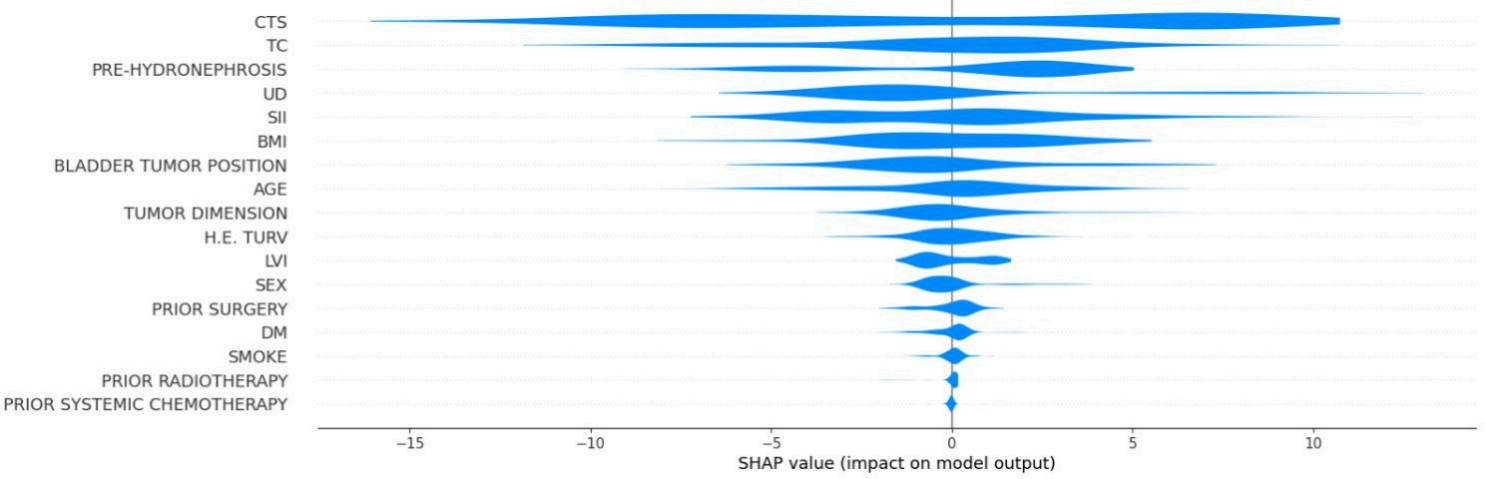
Violin plot of feature influence on disease-free survival prediction from the Shapley additive explanations (SHAP) analysis. CTS: clinical tumor stage; DM: diabetes mellitus; H.E. TURV: histological examination for transurethral resection of the bladder; LVI: lymphovascular invasion; PRE-HYDRONEPHROSIS: pretreatment hydronephrosis; SII: systemic immune-inflammation index; TC: tumor classification; UD: urinary diversion type*.*

Figure 2 displays the SHAP summary plot for the DFS model, illustrating the distribution and direction of impact of each feature on the predicted DFS across all patients. Clinical tumor stage and tumor classification exhibited the widest distribution of SHAP values, confirming their dominant influence, where the predicted DFS substantially increased or decreased depending on their values. SII displayed a more balanced distribution, with both positive and negative effects depending on the value. In contrast, features such as prior treatment (eg, surgery, radiotherapy, and chemotherapy) and lifestyle factors (eg, smoking status and diabetes) had a SHAP distribution clustered near 0, indicating limited predictive power.

**Figure 3.**
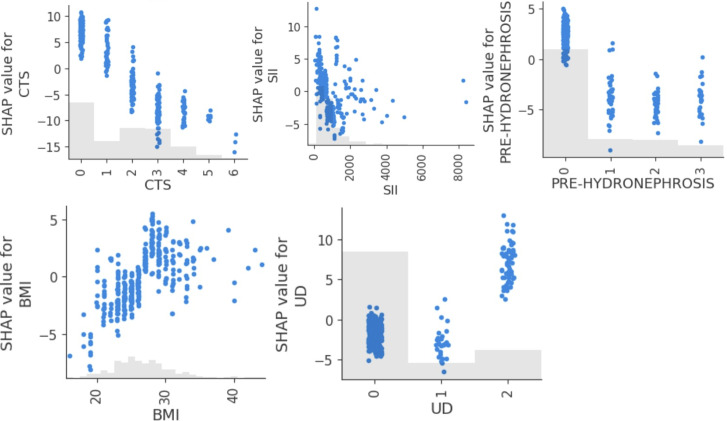
Shapley additive explanations (SHAP) scatterplots for the 5 most significant features influencing disease-free survival predictions (with BMI in kg/m^2^). CTS: clinical tumor stage (0: cTa, 1: cTis, 2: cT1, 3: cT2, 4: cT3, 5: cT4); PRE-HYDRONEPHROSIS: pretreatment hydronephrosis (0: no, 1: right hydronephrosis, 2: left hydronephrosis, 3: bilateral hydronephrosis); SII: systemic immune-inflammation index; UD: urinary diversion type (0: Bricker ileal conduit, 1: ureterocutaneostomy, 2: vesicoileal pouch)*.*

Figure 3 presents the SHAP dependence plots for 4 of the most influential features affecting DFS predictions. The x-axis shows the feature value, and the y-axis shows the SHAP value (ie, the impact on the model’s output). Clinical tumor stage showed a strong negative relationship with predicted DFS: as tumor stage increased, the SHAP values shifted sharply downward, indicating a consistent reduction in predicted DFS, aligning with the known prognostic role of tumor invasiveness in bladder cancer. SII demonstrated a nonlinear relationship, showing that patients with lower SII values had better SHAP values, while those with elevated SII showed increasingly negative impacts on DFS. This suggests a threshold effect, where systemic inflammation beyond a certain level contributes to poorer prognosis. The presence of pretreatment hydronephrosis had a negative impact on DFS prediction. Patients with low BMI had negative SHAP values, indicating reduced DFS, while those with moderate BMI experienced mildly negative predictions. At a BMI greater than 28, the SHAP values became positive, suggesting a potential protective effect exerted by higher BMI. Regarding the type of urinary diversion, vesicoileal pouch construction showed positive SHAP values, while other approaches had negative SHAP values.

### OS Prediction

**Figure 4.**
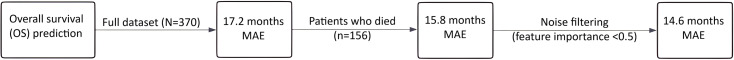
Progressive improvement in model accuracy. MAE: mean absolute error.

For OS prediction, the CatBoost model achieved an MAE of 17.2 months across the entire patient cohort. When the analysis was restricted to the subgroup of patients who had died (n=156), the prediction error improved to 15.8 months. After filtering features by importance, using a threshold of <0.5, the MAE further improved to 14.6, suggesting that a more compact feature set may improve predictive efficiency without compromising accuracy (Figure 4). This final model was selected for interpretation, as it maintained accuracy while reducing complexity.

**Figure 5.**
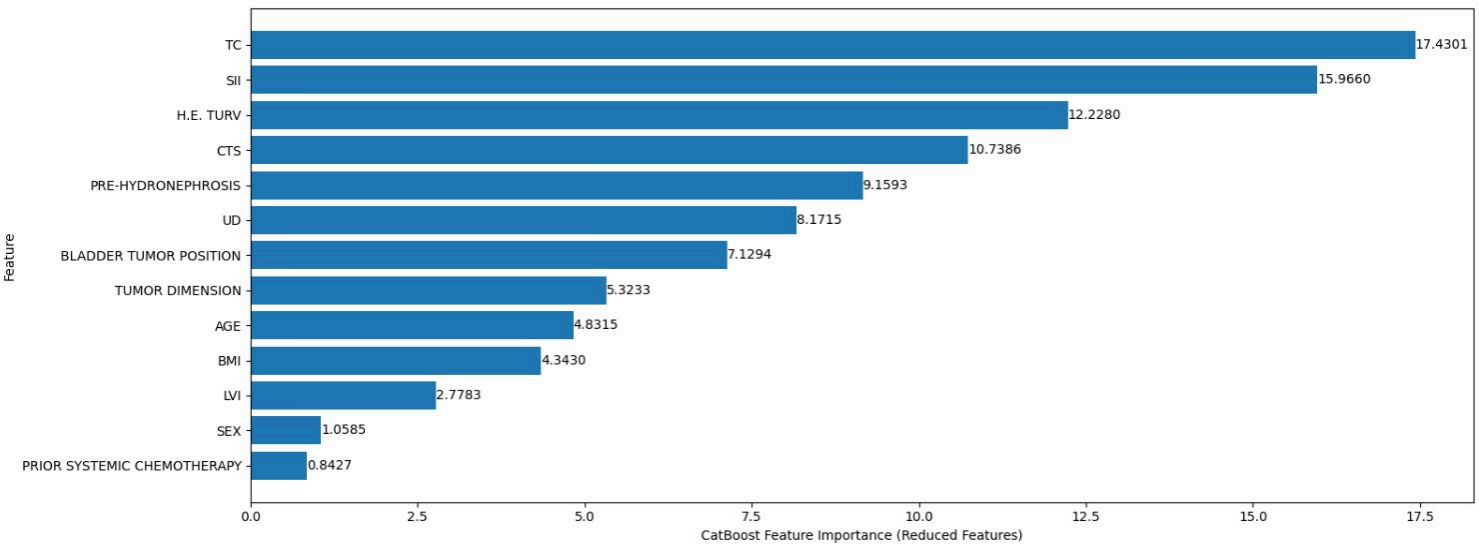
CatBoost feature importance ranking for the best-performing overall survival prediction model. CTS: clinical tumor stage; H.E. TURV: histological examination for transurethral resection of the bladder; LVI: lymphovascular invasion; PRE-HYDRONEPHROSIS: pretreatment hydronephrosis; SII: systemic immune-inflammation index; TC: tumor classification; UD: urinary diversion type.

Figure 5 presents the CatBoost feature importance ranking for the best-performing OS prediction model. Tumor classification emerged as the most influential predictor of OS in the final model with a value of approximately 17.5. SII followed closely with a value of approximately 15.5, highlighting the role of systemic inflammation in cancer progression and survival outcomes. The third feature was represented by histological findings (H.E. TURV). Other influencing factors included clinical tumor stage, pretreatment hydronephrosis, type of urinary diversion, BMI, and age.

**Figure 6.**
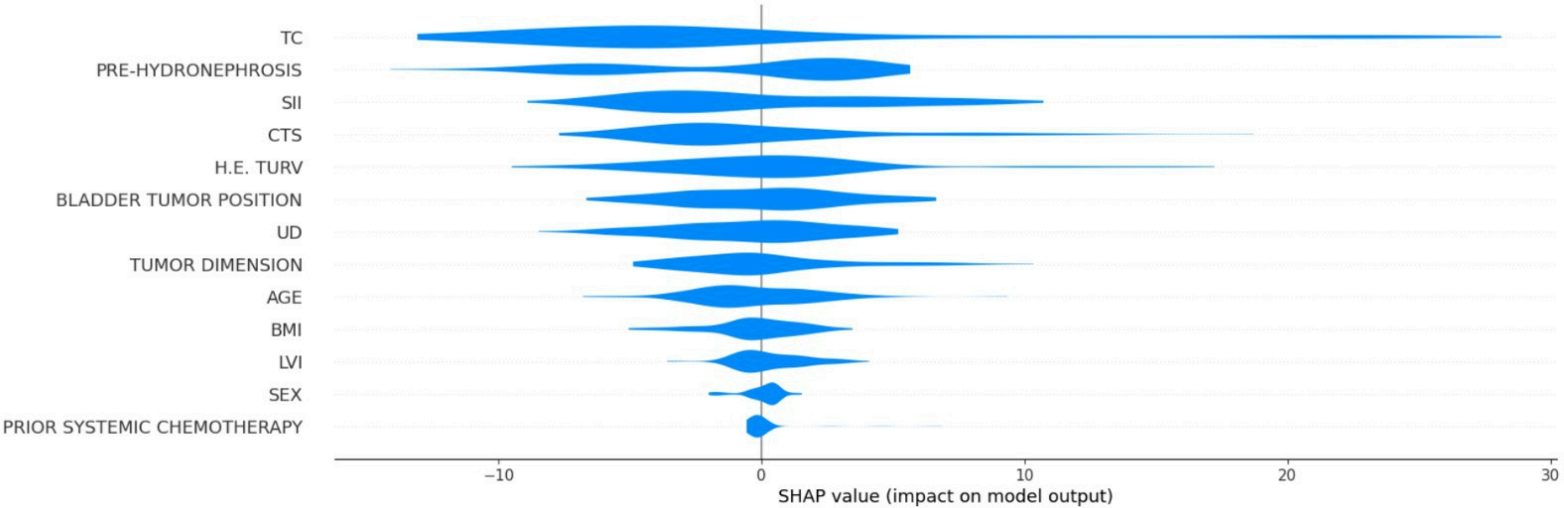
Shapley additive explanations (SHAP) violin summary plot for the final overall survival prediction model. CTS: clinical tumor stage; H.E. TURV: histological examination for transurethral resection of the bladder; LVI: lymphovascular invasion; PRE-HYDRONEPHROSIS: pretreatment hydronephrosis; SII: systemic immune-inflammation index; TC: tumor classification; UD: urinary diversion type.

Figure 6 displays the SHAP summary plot for the final OS prediction model. As expected, the most impactful variable was tumor classification, which showed a broad distribution. Pretreatment hydronephrosis and SII exhibited a wide SHAP distribution. Clinical tumor stage and histological findings (H.E. TURV) showed a similar overall effect on prognosis.

**Figure 7.**
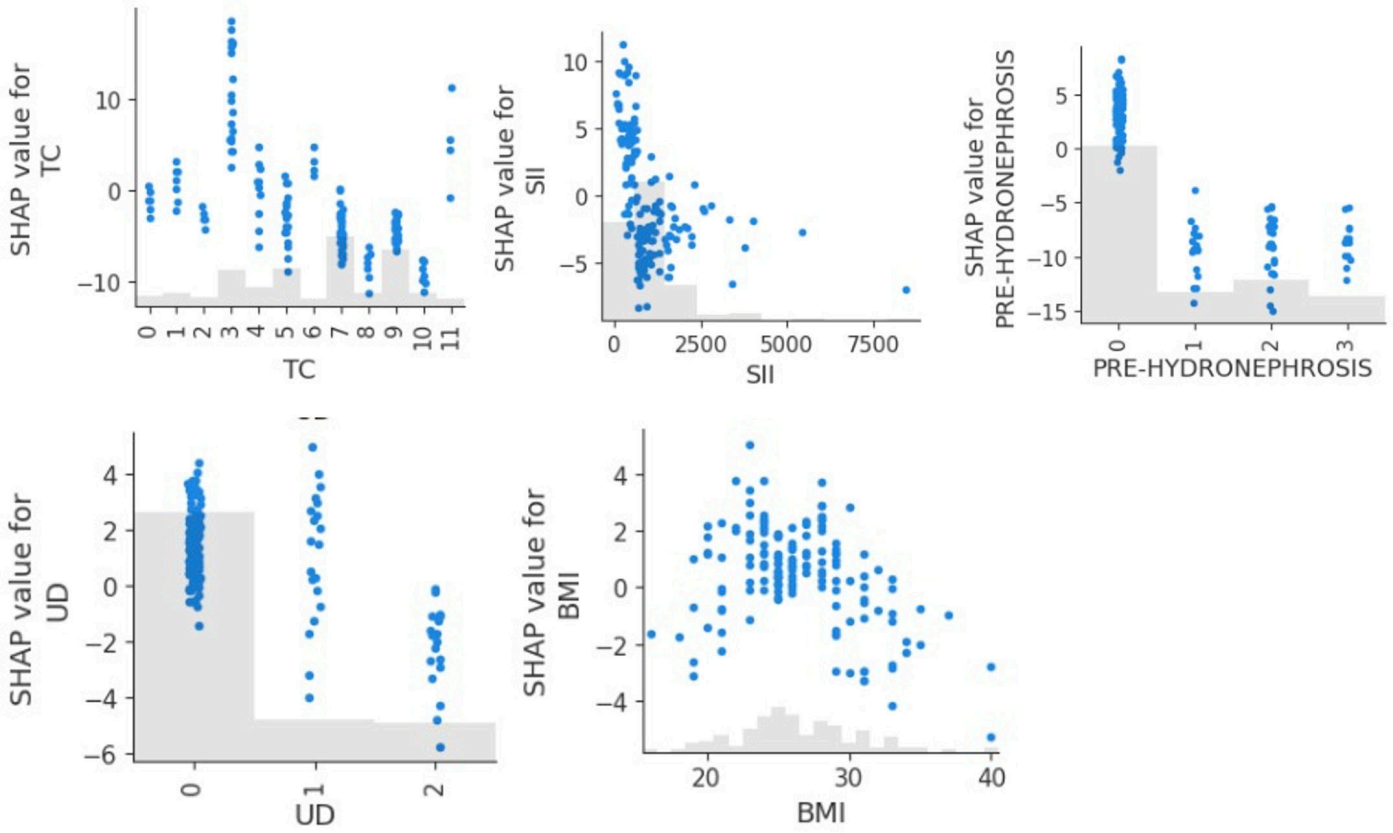
Shapley additive explanations (SHAP) scatterplots for the 5 most influential features influencing overall survival prediction (with BMI in kg/m^2^). PRE-HYDRONEPHROSIS: pretreatment hydronephrosis (0: no, 1: right hydronephrosis, 2: left hydronephrosis, 3: bilateral hydronephrosis); SII: systemic immune-inflammation index; TC: tumor classification (1: T0, 2: Ta, 3: Tis, 4: T1, 5: T2a, 6: T2b, 7: T3a, 8: T3b, 9: T4a, 10: T4b); UD: urinary diversion type (0: Bricker ileal conduit, 1: ureterocutaneostomy, 2: vesicoileal pouch).

Figure 7 presents the SHAP dependence plots for 5 key features influencing OS predictions. The tumor classification SHAP values indicated that patients with in situ cancers (value 3) had the best OS prediction, which gradually declined as the tumor stage advanced. SII showed a threshold effect, where predictions remained relatively stable up to a value of approximately 1000, then fell sharply, indicating that elevated inflammation is associated with a poor overall outcome. Pretreatment hydronephrosis was strongly linked to reduced predicted OS, where patients with this condition had uniformly negative SHAP values, not influenced by bilaterality. At the same time, BMI demonstrated a nonlinear pattern, where patients with very low BMIs had reduced OS predictions, moderate BMIs were associated with better outcomes, and the SHAP values began to decline again at higher BMI values, suggesting that both underweight and obesity may be associated with increased mortality risk in this population. The type of urinary diversion showed a different impact than that observed in DFS prediction, with vesicoileal pouch construction being associated with a lower OS.

### Cause-of-Death Classification

**Figure 8.**
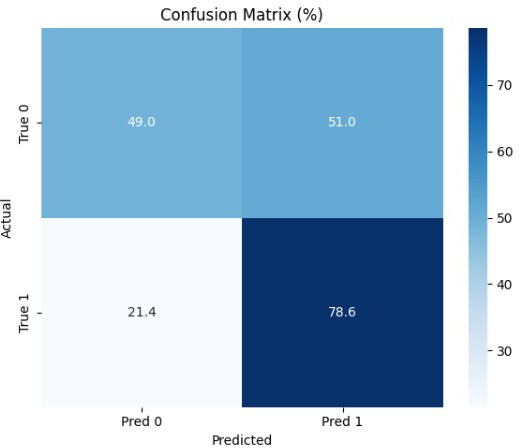
Confusion matrix for cause-of-death classification.

The CatBoostClassifier was trained to predict whether a patient’s death was tumor related. Due to class imbalance, only 14 of 78 deaths were cancer related; custom class weights and a reduced decision threshold of 0.12 were applied to maximize recall and minimize false negatives. The final model achieved a recall of 78.6% (Figure 8), correctly identifying 11 of 14 tumor-related deaths. The overall *F*_1_-score for the positive class was 0.44, with a precision of 31%. The model prioritizes sensitivity over specificity.

**Figure 9.**
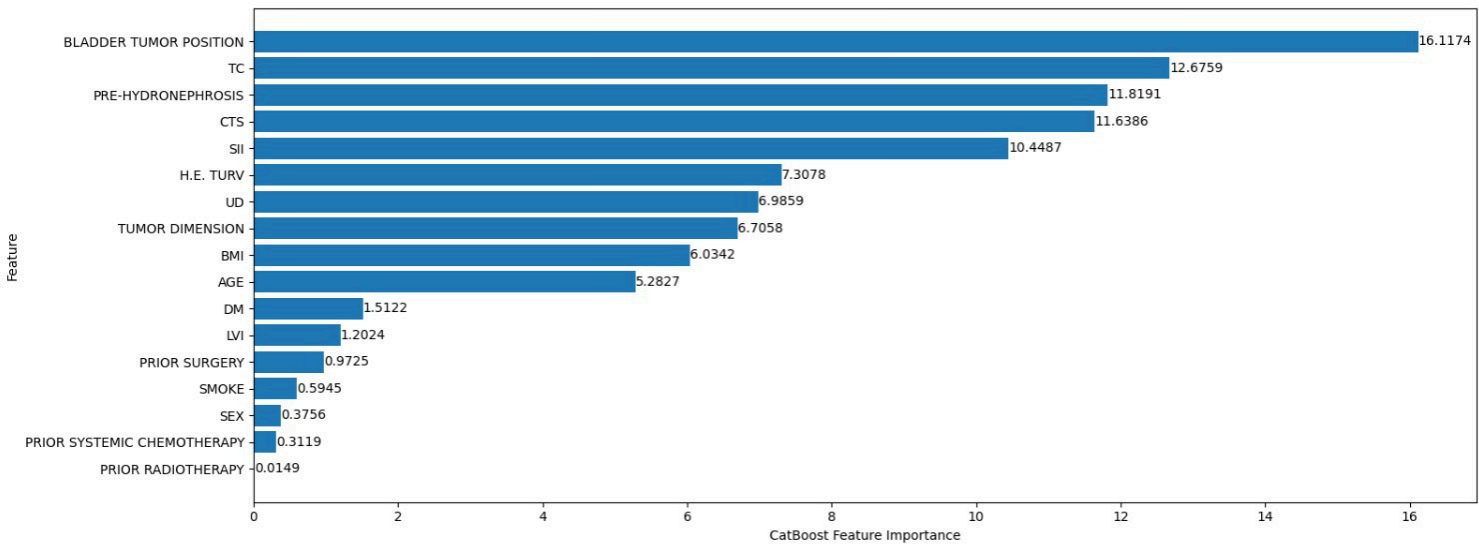
CatBoost feature importance ranking for the cause-of-death classification. CTS: clinical tumor stage; DM: diabetes mellitus; H.E. TURV: histological examination for transurethral resection of the bladder; LVI: lymphovascular invasion; PRE-HYDRONEPHROSIS: pretreatment hydronephrosis; SII: systemic immune-inflammation index; TC: tumor classification; UD: urinary diversion type.

Figure 9 represents the CatBoost feature importance ranking for the death cause classification model. The most influential feature was the anatomical position of the bladder tumor, with a value of approximately 16.5. This was followed by tumor classification and pretreatment hydronephrosis, both indicators of disease severity and progression.

Other key features included the clinical tumor stage and SII, with values of approximately 11 and 11.5, respectively.

**Figure 10.**
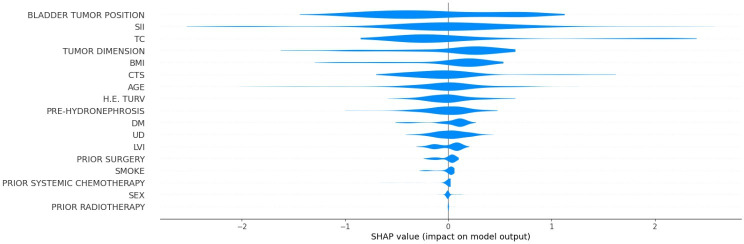
Shapley additive explanations (SHAP) summary plot for the tumor-related death classification model. CTS: clinical tumor stage; DM: diabetes mellitus; H.E. TURV: histological examination for transurethral resection of the bladder; LVI: lymphovascular invasion; PRE-HYDRONEPHROSIS: pretreatment hydronephrosis; SII: systemic immune-inflammation index; TC: tumor classification; UD: urinary diversion type.

**Figure 11.**
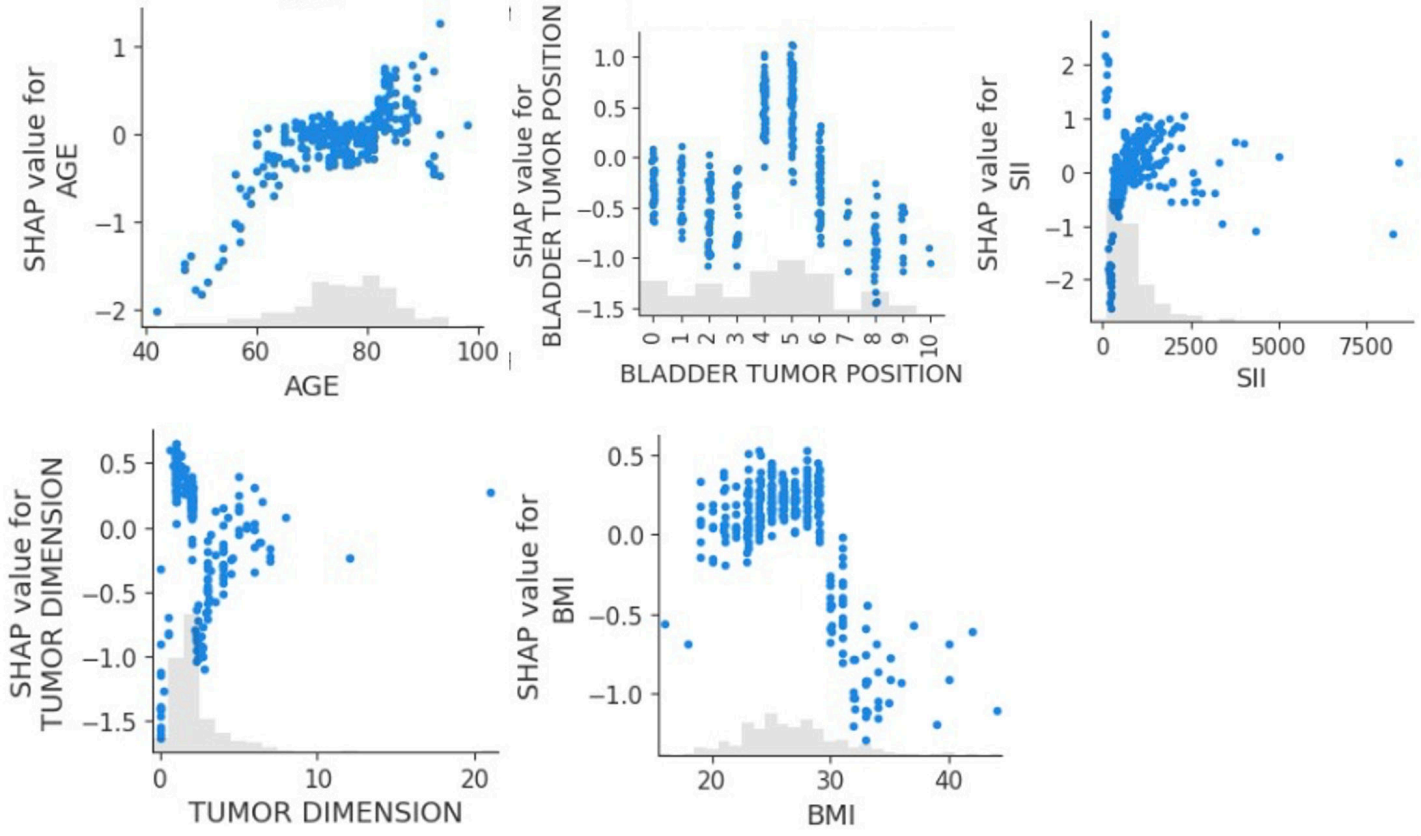
Shapley additive explanations (SHAP) scatterplots for the 5 most influential features influencing the tumor-related death classification model (with BMI in kg/m^2^, AGE in years, and TUMOR DIMENSION in cm; bladder tumor position: 0: intertrigonal zone, 1: right periosteal, 2: left periosteal, 3: dome, 4: posterior wall, 5: right lateral wall, 6: left lateral wall, 7: prostatic urethra, 8: anterior wall, 9: entire bladder, 10: bladder base). SII: systemic immune-inflammation index.

Figure 10 displays the SHAP summary plot for the tumor-related death classification model. The feature with the widest and most impactful distribution was bladder tumor position, which is addressed in detail in the discussion of Figure 11. SII was also influential, with positive and negative SHAP values. Tumor classification, pretreatment hydronephrosis, and clinical tumor stage generally acted to slightly decrease the predicted risk of tumor-related death for most patients, with little variability in their effect.

Figure 11 presents the SHAP dependence plots for 5 key features influencing the tumor-related classification model: age, bladder tumor position, SII, tumor dimension, and BMI.

The scatterplot for age exhibited a clear positive trend, showing that as patient age increased, the SHAP value for age also increased, indicating that older age consistently increased the predicted risk of tumor-related death. Bladder tumor position was another influential predictor in the tumor-related death classification model, highlighting that tumors located in the posterior wall and right lateral wall were more likely to be the cause of death. In contrast, tumors located in the anterior wall and at the bladder base or those spreading throughout the entire bladder were less likely to be the cause of death. Increasing SII and tumor dimension also had a moderate predictive value, respectively increasing and decreasing the probability that the patient’s cause of death was cancer. Finally, patients with a higher BMI showed a higher likelihood that the cause of death was cancer.

## Discussion

### Principal Findings

This paper presents the development of a survival prediction model using machine learning approaches for patients with bladder cancer. Our findings demonstrate that modern predictive algorithms show promising accuracy in forecasting DFS, OS, and cause of death. The limited sample size and the paucity of included categories for the analysis suggest that predictive algorithms trained with additional data and variables significantly improve the demonstrated accuracy.

The observation that age showed a positive correlation with survival outcomes is particularly intriguing and seemingly counterintuitive. This “age paradox” has been previously described in bladder cancer and other oncological settings and may reflect a combination of selection bias and underlying tumor biology rather than a true protective effect of age, which can be explained by several factors. Older patients often receive more conservative treatment, which potentially leads to selection bias in surgical candidates. Additionally, younger patients with bladder cancer have been reported to present more frequently with aggressive disease variants, which could account for their relatively poorer outcomes despite younger age [11]. These results align with recent systematic reviews and meta-analyses on radical cystectomy, which consistently report high complication rates and significant variability in postoperative survival across risk profiles [12]. Importantly, as this study is observational, the association between age and survival should be interpreted cautiously and not as a causal relationship.

Clinical tumor stage was the strongest predictor, which aligns with established prognostic factors in bladder cancer [13]. Additionally, inflammatory markers, particularly SII, showed a negative correlation with survival outcomes, supporting recent findings in other malignancies [14]. This relationship likely reflects the complex interplay between systemic inflammation and cancer progression, where elevated SII indicates a protumoral inflammatory state [15]. Our findings on systemic inflammatory indices are consistent with recent data indicating that platelet-to-lymphocyte ratio, systemic inflammation response index, pan-immune-inflammation value, SII, and neutrophil-to-lymphocyte ratio are associated with adverse outcomes in non–muscle-invasive bladder cancer [16].

SHAP analysis revealed a clear, monotonic decline in predicted survival with advancing clinical tumor stage, reinforcing its primary prognostic role in both DFS and OS. SII, by contrast, demonstrated a threshold effect where values above approximately 1000 were associated with a sharp drop in predicted DFS, suggesting a nonlinear relationship between systemic inflammation and patient outcomes. The observed association between urinary diversion type and survival outcomes should be considered exploratory. Previous studies had found that orthotopic neobladder reconstruction had a protective effect against urethral recurrence in male patients undergoing radical cystectomy for bladder cancer [17]. While this effect was not observed in our dataset, we found a positive association between vesicoileal pouch construction and improved survival. This association may reflect both patient selection and potential physiological advantages of this diversion type; however, given the limited number of patients within each diversion subgroup, it should be interpreted cautiously. Confounding factors such as surgical expertise and patient characteristics, which were not accounted for in the present study, may have influenced these findings. BMI showed a similar intriguing relationship: patients with an unhealthy BMI, either high or low, showed poorer outcomes; this may be related both to tumor characteristics and the surgical approach being limited in terms of radicality. This U-shaped association between BMI and survival was consistently observed across both DFS and OS outcomes, with moderate BMI ranges correlated with more favorable SHAP values. The findings support a metabolic vulnerability in patients with underweight as well as obesity, which may influence recovery or treatment tolerance.

Our machine learning models achieved prediction accuracies comparable to those reported in previous studies. The accuracy in cause-of-death prediction, although modest, represents an encouraging level, given the limited resources and the paucity of categories considered for the analysis, particularly when compared with studies published a few years ago that used significantly larger samples yet achieved marginally higher accuracy in mortality and recurrence prediction [18]. A recently published systematic review investigating machine learning algorithms for bladder cancer cystectomy outcomes found that most of the algorithms did not exceed 70% accuracy and, in some cases, performed with approximately 60% accuracy [19]. The integration of SII into predictive models represents an auspicious direction. As a low-cost, readily available biomarker, SII could enhance current prognostic tools without adding significant complexity or cost to patient evaluation [14].

A notable limitation of our study is the relatively high MAE in survival predictions. These MAE values render the algorithm unsuitable for precise individual patient counseling or treatment planning where accurate timing is critical, such as in emergency settings or for patients exhibiting postoperative complications [20]. However, this level of accuracy remains acceptable for clinical trial patient stratification and allocation, particularly in trials where broad risk categories rather than precise survival estimates are needed for randomization. Such applications include balancing treatment arms in clinical trials by identifying comparable risk groups or supporting enrollment decisions in competing risk analysis, where precise timing is less critical than overall risk assessment [21].

### Limitations and Reproducibility

This study is subject to some limitations when interpreting the results. The relatively limited dataset size (N=370 initially; reduced to 312‐347 for specific analyses) inherently constrains the generalizability and robustness of the developed models. While machine learning algorithms such as CatBoost are robust on smaller datasets, their predictive power can be substantially enhanced with larger cohorts.

Secondly, the monocentric nature of the data collection, originating solely from Fondazione Policlinico Universitario Agostino Gemelli IRCCS in Rome, Italy, introduces a potential for selection bias and limits external validity. Patient characteristics, treatment protocols, and population demographics can vary significantly across institutions and geographical regions. The findings from this study may not be directly transferable to other clinical settings without further validation on diverse, external datasets.

Thirdly, while rigorous data cleaning was performed, the inherent human factors associated with retrospective data extraction from medical records cannot be eliminated.

### Conclusion

Our study demonstrates the potential utility of machine learning approaches in predicting bladder cancer outcomes following cystectomy. While the achieved accuracy levels are modest, they align with current literature benchmarks and provide a foundation for future development. The identification of clinical tumor stage as the primary predictor, along with the consistent negative correlation of SII with survival outcomes, validates these parameters as valuable prognostic indicators. In particular, the SHAP analysis revealed a monotonic decline in predicted DFS and OS with advancing clinical tumor stage, reaffirming its role in risk stratification. On the other hand, SII exhibited a threshold effect, where values above approximately 1000 were associated with a rapid drop in predicted survival, reinforcing the adverse prognostic impact of systemic inflammation. The current model’s performance, though not suitable for precise individual prognostication, shows particular promise for clinical trial stratification and cohort allocation. Future studies with larger datasets and additional predictive variables may enhance the model’s accuracy and broaden its clinical applications. Integrating readily available biomarkers, such as SII, represents a cost-effective approach to improving prognostic tools. These findings contribute to the growing body of evidence supporting the role of machine learning in oncological decision-making while acknowledging the need for continued refinement and validation in larger cohorts.

## Abbreviations

AI: artificial intelligence
DFS: disease-free survival
MAE: mean absolute error
OS: overall survival
SHAP: Shapley additive explanations
SII: systemic immune-inflammation index
TRIPOD: Transparent Reporting of a Multivariable Prediction Model for Individual Prognosis or Diagnosis
